# An Account of the Regular Gradation in Man, and in Different Animals and Vegetables; and from the Former to the Latter: Illustrated with Engravings Adapted to the Subject

**Published:** 1799-06

**Authors:** 


### CRITICAL RETROSPECT
### OF
### MEDICAL AND PHYSICAL LITERATURE.

### [FOREIGN AND DOMESTIC.]

### COMPARATIVE ANATOMY.

*An Account of the Regular Gradation in Man, and in different Animals and Vegetables; and from the former to the latter: illuſtrated with engravings adapted to the ſubject.* By CHARLES WHITE. Read to the Literary and Philoſophical Society of Mancheſter, at different meetings, in the year 1795. Quarto, 146 pages text, and ſeven ſheets appendix. Three plates folio, and one quarto, 1799, (Price 10s. 6d.) *London.* Dilly.

This claſſical volume the author introduces with a modeſt advertiſement, in which, among other profeſſions teſtifying the candour and liberality of his ſentiments, he requeſts the phyſiologiſt, " not to conſider the preſent eſſay as a complete treatiſe on the ſubject, but only as a collection of hints and obſervations for the uſe of others who have leiſure and opportunity to carry on the inveſtigation."

The whole is divided into four parts; in the firſt of which the author treats of *gradation in general,* and after having made ſome ingenious obſervations on the genus of animals called *canis* by naturaliſts; on the *veſpertilio* or bat; on the *lemur*, or maucauco; on the *ſimiæ*, or apes; on the circumſtance of America being inhabited by a race of animals unknown to the other Continent; on birds and their faculties or energies compared with the ſtructure of their ſkulls and beaks—he draws the following inferences from the facts and obſervations before detailed:

" That there is a general gradation from man through the animal race; from animals to vegetables, and through the whole vegetable ſyſtem. By gradation, I mean the various degrees in the powers, faculties, and organization. The gradation from man to animals is not by one way; the perſon and actions deſcend to the orang-outang, but the voice to birds, as has been obſerved.

" That there are many quadrupeds, inſects, birds, and fiſhes, which appear to be created for particular climates, and cannot live in any other.

" That many animals and vegetables exiſt in the old world, which were not found in the new one, when diſcovered by *Columbus*; and that there are many animals and vegetables found in the new world, which were never known in the old.

" Laſtly, that thoſe animals which were common to both worlds, were only to be met with in the northern hemiſphere, in which the new and old world had probably communications near the north pole. Theſe animals were about twenty-ſix in number."

In the ſecond part of his work, the author treats of the *gradation of man in particular*; of the gradation of bones; the length of the ulna in ſkeletons; the length of the fore-arms of living Negroes and Europeans; the ſkulls of different nations; the facial line; the gradation of the cartilages, muſcles, tendons, ſkin, hair, ſweat, catamenia; the rankneſs of ſmell; the degrees of ſenſible heat; the duration of life; the ſize of the penis, teſtes, ſcrotum, &c. &c. and from theſe multifarious reflections he deduces the following concluſions:

" 1. That

"1. That there are material differences in the corporeal organization of various classes of mankind.

"2. Taking the European man as a standard of comparison, on the one hand, and the tribe of simiæ on the other; and, comparing the classes of mankind with the standards, and with each other, they may be so arranged as to form a pretty regular gradation, in respect to the differences in the bodily structure and economy, the European standing at the head, as being farthest removed from the brute creation.

"3. That the African, more especially in those particulars in which he differs from the European, approaches to the ape.

"4. That the following characteristics, which distinguish the African from the European, are the same, differing only in degree, which distinguish the ape from the European:

*In the System of the Bones.*

"The narrow and retreating forehead and hind-head; the flat bone of the nose; the great distance betwixt the nose and mouth; the small retreating chin; the facial line; the great distance betwixt the ear and the forepart of the mouth; the small distance between the *foramen magnum* and the back of the head; the long and strong under jaw; the large bony sockets which contain the eyes, and the wide *meatus auditorius*; the long fore-arm; the flat foot, and the length, breadth, shape and position of the *os calcis*.

*In other parts of the System.*

"The broad and flat cartilage of the nose; the small *gastra-cnemii*, and large temporal muscles; the long *tendo achillis*; the thick skin and short woolly hair; the small brain; the long breasts of the females; the parts of generation; the paucity of different discharges; the rank smell; their manner of walking; the power of adaptation to a warm climate; their shorter period of life.

"5. That different classes of men are not liable to all the diseases incident to mankind, and that they are infested with different insects.

"6. That, in comparing the classes of mankind with each other, and with the brute creation, as in the second article, there is a gradation also discoverable in the senses of *seeing*, *hearing* and *smelling*, in *memory*, and in the powers of *mastication*, but in a contrary order to that above stated, the European being least perfect, the African more so, and the brutes most perfect of all, in these particulars."

In the third part of the work, the author takes a comparative view of the *hair* of different nations, and the wool of animals, in different climates; and after observing that the hair in the human race was the same 1800 years ago as it is at present, concludes this section with the following result: "Perennial hair, annual hair, and wool, seem to be three distinct productions, essentially different from each other. Of the perennial hair, there are various species; as that upon the head of an European; that upon the chin; and other parts of the body. The hair of the negro's head seems to be a different species from the European hair, and not a variety occasioned by any difference of climate, or from any peculiar mode of living, dependent on their want of civilization."

In the fourth and last part of the work, Mr. White speaks, at some length, *of the colour or complexion of man*. This highly-interesting part of the subject, the author concludes with the following animated observations:

"Different species of men being once admitted, it will become a proper object of physiological inquiry to determine their number and distinction, with the merits, excellencies, and defects of each. In pursuing this inquiry, there there is no doubt but gradation will afford the proper clue to direct us. What the number of species may be, is not perhaps easy to determine. The four quarters of the globe will each, probably, furnish us with at least one. In Africa, however, there seems to be more than one species; and perhaps the lowest degree of the human race resides there. I am inclined to think that hair, rather than colour, ought to guide us in that quarter; and that it is not the blackest inhabitants, but those with extremely short hair, and a most ungracious appearance, as the Hottentots, who may be reckoned the lowest on the scale of humanity. The Negro, the American, some of the Asiatic tribes, and the European, seem evidently to be different species.

"Ascending the line of gradation, we come at last to the white European; who, being most removed from the brute creation, may, on that account, be considered as the most beautiful of the human race. No one will doubt his superiority in intellectual powers; and I believe it will be found, that his capacity is naturally superior to that of every other man. Where shall we find, unless in the European, that nobly arched head containing such a quantity of brain, and supported by a hollow conical pillar, entering its centre? Where the perpendicular face, the prominent nose, and round projecting chin? Where that variety of features and fulness of expression; those long, flowing, graceful ringlets; that majestic beard; those rosy cheeks and coral lips? Where that erect posture of the body and noble gait? In what other quarter of the globe shall we find the blush that overspreads the soft features of the beautiful women of Europe; that emblem of modesty, of delicate feelings, and of sense? Where that nice expression of the amiable and softer passions in the countenance; and that general elegance of features and complexion? Where, except on the bosom of the European woman, two such plump and snowy white hemispheres tipt with vermillion?"

In an '*Appendix*' the author has enriched this valuable publication with '*Detached Passages selected from Professor Soemmering's Essay on the Comparative Anatomy* (*Corporeal Difference* says the German title) *of the Negro and European*;" translated by Dr. Holme. The whole is concluded with a few 'Notes,' by the author.

